# The effects of combined endurance training of different intensities and resistance training on bone mineral density, microstructure and mechanical properties of rats

**DOI:** 10.3389/fendo.2026.1716038

**Published:** 2026-01-26

**Authors:** Ting Mo, Xin Zhang, Yueming Zhao, Guangxin Li, Zhanjia Zhang, Shilun Hou

**Affiliations:** 1 School of Sports Medicine and Rehabilitation, Beijing Sport University, Beijing, China; 2 Department of Physical Education, Peking University, Beijing, China; 3 Key Laboratory of Exercise Rehabilitation Science of the Ministry of Education, Beijing Sport University, Beijing, China

**Keywords:** biomechanical, bone microstructure, bone mineral density, endurance and resistance training, puberty

## Abstract

**Objective:**

To explore the effects of different intensities of endurance training combined with resistance training on the bones of growing male rats, and to provide the optimal exercise plan for bone mineral accumulation in adolescents.

**Methods:**

Thirty 6-week-old male Sprague-Dawley rats were randomly divided into 5 groups: control (C), resistance training (R), low-intensity endurance + resistance (LR), moderate-intensity endurance + resistance (MR), and high-intensity interval + resistance (HR). After 8 weeks of exercise intervention, the bone mineral density, bone microstructure, bone mechanical properties and bone remodeling of rats were assessed. One-way analysis of variance (ANOVA) was used to test the results obtained by the detection methods.

**Results:**

The body weight of the exercise group was lower than that of the control group. The endurance and resistance training group (LR/MR/HR) had significantly higher bone mineral density than the control group (*p*’s < 0.05). There was no difference in bone metabolism markers among the groups. In the result of bone volume fraction, only the MR group was significantly higher than the control group (*p* = 0.03); the number of trabeculae showed statistical differences in the LR group and the MR group (*p*’s < 0.05). Each exercise group showed significantly higher maximum load and fracture stress than the control group (*p*’s ≤ 0.001), but no difference in maximum strain was shown.

**Conclusion:**

Combined endurance and resistance training improved bone mineral density and mechanical strength in growing male rats, with moderate-intensity endurance training showing the most consistent improvements in bone microarchitecture.

## 1 Introduction

Puberty is a critical period for bone mineral accumulation (1). At this time, bone mass accumulates rapidly (about 50% of peak bone mass is achieved), and about 90% of peak bone mass can be achieved by the end of adolescence (2). Adolescence is a key window to increase peak bone mass, and the accumulation of bone mass during this period helps to reduce the risk and severity of osteoporosis in later life (3). Epidemiological studies have confirmed that increased bone mineral density in childhood and adolescence can reduce the risk of fracture later in life (4), and peak bone mass achieved in adolescence and early adulthood is a strong predictor of osteoporosis in later years (5). Clark et al. (6) found that a one standard deviation reduction in bone mass during childhood increased the risk of adult fracture by 89% and emphasized that increasing bone mass during adolescence was as important for fracture prevention as preventing bone loss in old age. Therefore, it is critical to maximize bone mass accumulation early in life, especially during adolescence, which is more feasible than using resistance exercise to increase bone mass in later life (7, 8).

Exercise has well-documented beneficial effects on the mechanical properties of bone (9). Human research have shown that resistance exercise or endurance exercise can promote bone development in adolescents, as well as increase bone size and mineral content (10, 11). Moreover, animal research have shown that running is an effective method to improve bone mechanical performance, and appropriate intensity can increase bone mineral density and bone strength (12, 13). Eliakim et al. (14) found that endurance exercise such as running for 5 weeks increased markers of bone formation (such as osteocalcin, BALP) and decreased markers of bone resorption (such as NTX) in male adolescents. However, compared with running, studies have shown that resistance training may increase bone mass more effectively by accelerating cortical drift and stimulating bone formation, which is an effective exercise type to promote bone mass accumulation during growth period (15). Michael (16) pointed out that resistance training can increase bone mass and structural strength, improve tissue quality, and thus enhance bone fracture resistance in humans and mice. Several studies in children and adolescents have demonstrated the effectiveness of resistance training in promoting bone formation. Specifically, Quiterio (15) identified resistance training as a key lifestyle factor for achieving optimal peak bone mass. The studies of Kobayashi (17), Dias (18), Blimkie (19), Nichols (20) and Almstedt (21) also consistently support the effect of resistance training on bone density gain in adolescents.

In order to explore a more effective way to gain bone mass, some studies have combined endurance and resistance training. Campos et al. (22) found that a combination of endurance and resistance training induced greater increases in bone mineral content in adolescents than endurance training alone. A study by Wang et al. (23) further confirmed that the bone mineral density of mice receiving 8 weeks of endurance training combined with resistance training was significantly higher than that of the control group undergoing either type of training alone, indicating a synergistic effect of combined training.

Although research has shown that endurance combined with resistance training has advantages over a single training mode, there is still controversy regarding the selection of endurance intensity in endurance combined with resistance training. The purpose of this study is to explore the optimal intensity combination of endurance training combined with resistance training to improve bone mineral density and bone biomechanical properties in male rats during adolescence. The findings of the current study would provide a most effective training method for teenagers in the future, which can maximize the increase of bone mineral density and bone mechanical properties.

## 2 Methods

### 2.1 Animals and sample collection

Thirty 6-week-old male SD rats, weighing 234.58 ± 7.37 g, were purchased from Beijing Vital River Laboratory Animal Technology Co., Ltd., with the qualification certificate (No. 110011241109713336) and animal experiment license (SCXK Jing 2021-0011). After arrival, the rats were housed in an isolated animal facility at the Animal Experiment Center of Beijing Sport University, with 3 rats in each cage. The rats were fed with the standard rodent feed provided by the laboratory animal center, which met the national standards, and had free access to food and water. The experimental protocol was approved by the Animal Ethics Committee of Beijing Sport University (Approval number: 2024337A) and was carried out in accordance with the regulations for the use of experimental animals. All animals were sacrificed at 48 hours after the last training, collected and stored for biological purposes to minimize any residual effects from the last training. The rats were fasted for 6 hours before the experiment. Rats were anesthetized using an animal gas anesthesia system. The anesthetic agent was isoflurane. First, induction was carried out in an induction chamber at a concentration of 3%, and after the rats were anesthetized, maintenance was performed at a concentration of 2%. After completing the dual-energy X-ray test and collecting the blood, the rats were sacrificed by abdominal aortic bleeding under deep anesthesia. Finally, the death of the rats was determined by checking whether their hearts were beating and whether their pupils had dilated. After confirming the death of the rats, the samples were collected.

Blood samples (≥5 mL) were collected from the abdominal aorta using a disposable medical syringe. After clotting at room temperature for 10–20 min, the samples were centrifuged at 3000 rpm for 15 min at 4°C to isolate serum. The right femur was fixed in 4% paraformaldehyde for Micro-CT analysis, while the left femur was wrapped in saline-soaked gauze and stored at -20°C for subsequent three-point bending testing.

### 2.2 Intervention methods and procedures

**Figure 1:**
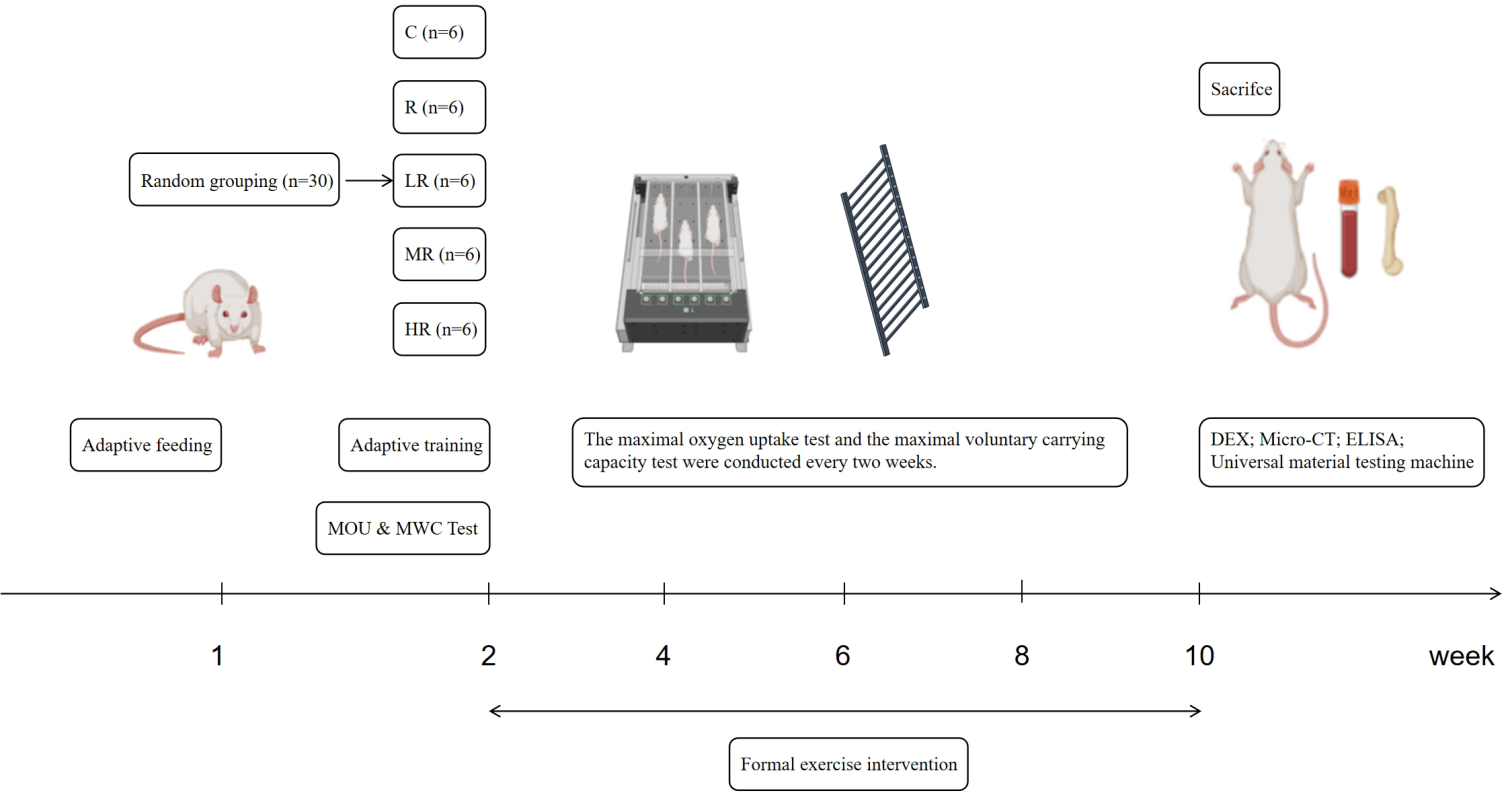
The experimental flow chart (by Biorender and Figdraw).

After one week of adaptive feeding, the rats were stratified and randomly divided into five groups based on their body weight: Control group (C, n = 6), resistance training (R, n = 6), low-intensity endurance + resistance training group (LR, n = 6), moderate-intensity endurance + resistance training group (MR, n = 6), and high-intensity interval + resistance training group (HR, n = 6). After grouping, there was no statistically significant difference in body weight among the groups. During the experiment, all the animals were allowed to freely consume the standard maintenance feed and water. The control group did not engage in any exercise, and the other groups were given different forms of exercise training for a total of 8 weeks. On Mondays, Wednesdays and Fridays, we performed resistance training. On Tuesdays, Thursdays and Saturdays, we performed endurance training. Each training session was conducted at the same time of the day. The experimental procedure is shown in **Figure 1**.

#### 2.2.1 Ladder-climbing exercise

Ladder-climbing training in rats is very similar to the motor parameters and physiological adaptations observed in human resistance training (24). In rats, extensive exercise based on ladder climbing activity has been shown to increase bone mineral density (25). Therefore, ladder climbing training will be used as the resistance training exercise program in this study. The ladder training was conducted on a 110cm high, 18cm wide, and 85-degree inclined ladder. At the top of the ladder, a dark box measuring 20cm*20cm*20cm was placed. On the tail of the rats, small containers with lead beads that could hold the weight were wrapped with white tape to simulate the weight-bearing movements of humans.

Adaptive training was performed for 3 days during the adaptive exercise week, and the rats were allowed to climb from the bottom to the top with no weight or with 10% to 20% of their body weight. Before the formal training and after 2, 4, 6, and 8 weeks of training, we tested the maximum load of the rats in each group. During the 8-week training period, we delimit the exercise load of the period according to the latest maximum load of the rats. During the formal training, the rats were initially placed at the bottom of the ladder. The tail stimulation was used to encourage the rats to climb up the ladder. Once they reached the top, the rats were allowed to rest in the simulation cage for 2 minutes, and this process was repeated 8 times.

##### 2.2.1.1 Maximum weight carried test

The results of the maximal weight carried test (MWC) were used as the basis for load control, and the load increasing intensity was 30%, 50%, 60%, and 70% MWC. The load was increased in the condition that the rats were able to perform 8 repeated climbs. The initial load was set at 75% of the body weight, and then 30 g of weight was added in successive increments until the maximum load was reached (26). The criterion of exhaustion was that the rat’s tail was still unable to climb the ladder after 3 consecutive stimuli, and there was a 60-second rest interval between each climb. When the MWC was applied to training, it was required to retest every 2 weeks, followed by test phases of 50%, 75%, 90%, and 100% of the previous MWC, with a 60-second rest interval between each climb. After that, 30 g weight was added in increments until a new MWC was determined, with a 120-s rest interval between each climb. MWC was completed in 4–9 sessions (27).

#### 2.2.2 Treadmill running exercise

The animal treadmill used in this study was a DSPT-208 model, equipped with five running lanes and an adjustable speed range of 0–70 m/min, and featured electrical stimulation as well as light and sound control switches. The VO_2_max of rats in each group was tested before the formal training and after 2, 4, 6, and 8 weeks of training, and the exercise load of each session was formulated based on the latest VO_2_max during the 8-week training period. Before the formal training begins, there will be 3 days of adaptive training. With the minimum running frequency and training duration, short-duration exercises of different speeds (5 minutes each or shorter) will be repeated to avoid heat shock or to achieve the effect of endurance training. After the formal training began, rats in the low-intensity group ran continuously at 40% to 50% VO_2_max for 60 min, and rats in the moderate-intensity group warmed up at 40% to 50% VO_2_max for 5 min, formally ran at 65% to 70% VO_2_max for 50 min, and finally ran at 40% to 50% VO_2_max for 5 min. Rats in the high-intensity interval group warm up at 50% to 60% VO_2_max for 5 min. During the formal training, the rats ran at 85% to 95% VO_2_max for 2 min, followed by a recovery run at 50% to 60% VO_2_max for 2 min, and performed 13 rounds (the last recovery run was directly included in the recovery run before the end). Finally, the run was resumed at 40% to 50% VO_2_max for 5 min.

##### 2.2.2.1 Maximal oxygen uptake test protocol

All rats underwent a maximal oxygen uptake test 48 h after the last familiarity with the treadmill. The rats were weighed before the test. Gas calibration was conducted before each test, using standard gas with an oxygen content of 20.50% and a carbon dioxide content of 0.50% to ensure the accuracy of the test. The experiment utilized the gas metabolism monitoring system (Oxymax Equal Flow) from the American Columbus Instrument Company, the modular treadmill (Modular Treadmill), and the rat metabolism monitoring system (Oxymax). The treadmill running speed was increased at a rate of 5 m/min every 3 min until the maximum VO_2_ speed was reached [oxygen consumption (VO_2_) was established when the plateau was reached despite the increase in running speed (28)]. The platform speed at VO_2_max was taken as the maximum speed (Vmax) and used to formulate exercise training prescriptions (29–31). For instance, when the rat’s oxygen uptake reaches a plateau (e.g., 40 ml/kg/min) and no longer increases with further speed increments, the corresponding treadmill speed (e.g., 30 m/min) is recorded and defined as the running speed at VO_2_max. Subsequent training regimens of different intensities are then individually prescribed based on a percentage of this speed (e.g., moderate intensity corresponding to 65%–70%).

### 2.3 Outcome measures

#### 2.3.1 Bone mineral density measurements

After the rats were anesthetized, they were placed prone and scanned under a dual-energy X-ray absorptiometry platform (Lunar iDxa, GE, USA). The density of the right femur of the rats *in vivo* was measured using the computer medium and small animal model.

#### 2.3.2 Bone microstructure indexes

The femur sample was removed from the fixative, the excess fluid was dried with gauze, and the sample was placed in the instrument scanning bed to start scanning, after which the original image was obtained. The right femur of rat was scanned using the micro-CT system of NMC-200 with a maximum resolution of 7.5 μm, and the scanning parameters were: 1. Operating voltage: 80 KV, current: 0.06 mA; 2. Radial field of view: lateral FOV: 51mm, axial FOV: 16.014mm; 3. Scanning speed: the fastest is 4 s/bed; 4. Thresholds: 1642. The scanning site was located at 1 mm from the femoral growth plate, and the original images were reconstructed by 3D reconstruction software Recon. The data analysis software Avatar was used to analyze the ROI of the target region, and the same region was selected for all samples for analysis to obtain the required parameter values and export the data.

We analyzed the following data from the femur: Bone volume parameters (BV/TV, %), trabecular density (BMD, mg/cm^3^), trabecular number (Tb. N, 1/mm), trabecular thickness (Tb. Th, mm), trabecular separation (Tb. Sp, mm), structural model index (SMI), trabecular connection density (Conn. D,1/mm^3^).

#### 2.3.3 Three-point bending mechanical testing

At room temperature, the femur was removed after the rats were sacrificed and the excess muscle tissue was removed and placed on the experimental support of the microcomputer controlled electronic universal material testing machine (UTM5305). The span was set to 20 mm, and the position of the bone was: The indenter above the testing machine can be pressed to the position of the middle part of the femur at each beginning of the detection of downward pressure. The descending speed of the indenter of the testing machine is set to downward 2 mm/min, and the experiment is completed when the indenter drops to the femur after the beginning of the experiment. Maximum load, fracture stress and maximum strain were measured.

#### 2.3.4 Serum-related indexes

Before the rats were sacrificed, the rats were kept under general anesthesia, and at least 5 ml of blood was drawn from the abdominal aorta using a disposable 5 mL syringe and transferred into vacuum tubes containing separation gel. The blood was allowed to stand for 10–20 min, then placed into a high speed refrigerated centrifuge and centrifuged at 3000rpm (13.3°C) for 15 min to obtain serum samples. The serum levels of osteocalcin (OC) and β-collagen degradation products (β-CTX) related to bone formation and resorption were calibrated by enzyme-linked immunosorbent assay (ELISA Elabscience Biotechnology Co., Ltd.).

### 2.4 Statistical analysis

Data were analyzed by SPSS (IBM SPSS Statistics 26). Shapiro-Wilk Test was used to test the normal distribution of data, and “mean± standard deviation” (mean ± SD) was used for statistical description of data following normal distribution. Median (interquartile range) [M(Q)] was used to describe the data that did not follow the normal distribution (skewed distribution). The test results were compared using one-way ANOVA or Kruskal-Wallis H test for independent samples (not following normal distribution). If the variance was homogeneous, the result of ANOVA test was the final result. If the variance was not uniform, the result of Welch and Brown-Forsyth test was used as the final result. The Bonferroni *post-hoc* test was used to further analyze the data. *p* < 0.05 was considered statistically significant. GraphPad Prism 9 was used for further statistical analysis and data visualization.

## 3 Results

### 3.1 Different forms of exercise can reduce the weight and body fat percentage of rats

**Figure 2:**
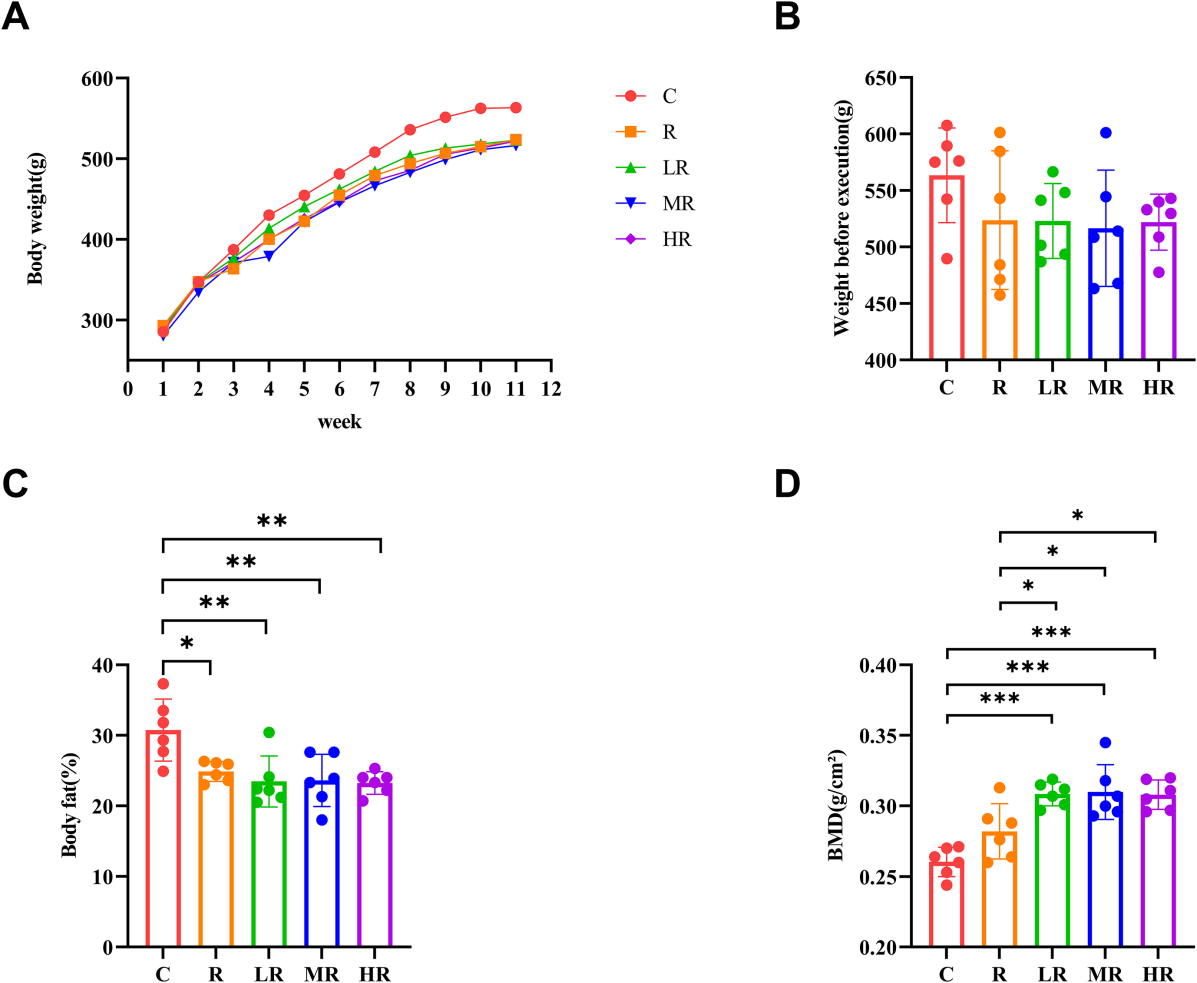
**(A)** Weight changes of rats during the experiment. **(B)** The effect of 8-week exercise intervention on the body weight of rats. **(C)** Comparison of body fat percentage among different groups of rats. **(D)** Comparison of bone density of the right femur of different groups of rats (*indicates *p*<0.05; **indicates *p*<0.01; ***indicates *p*<0.001).

The weight of the rats was recorded every week, as shown in **Figure 2A**, reflecting the weight change of the rats during exercise. During exercise, the weight of the rats in each group maintained a steady upward trend, but the weight of the rats in the control group was higher than that in the exercise group from the second week of training. After an eight-week exercise intervention, although the weight of the exercise group decreased, there was no statistical difference (*p*>0.05) (**Figure 2B**). However, the body fat percentage of the exercise group was significantly different from that of the control group (η^2^ = 0.487, *p* = 0.002, F = 5.936; R vs C, *p* = 0.038; LR vs C, *p* = 0.006; MR vs C, *p* = 0.007; HR vs C, *p* = 0.004) (**Figure 2C**).

### 3.2 The effects of different forms of exercise on bone mineral density

DEX test results showed that after eight weeks of training, the LR, MR and HR groups had significantly higher bone mineral density of the right femur than the C and R groups (η^2^ = 0.689, *p*<0.001, F = 5.936; LR vs C, *p* < 0.001; LR vs R, *p* = 0.04; MR vs C, *p* < 0.001; MR vs R, *p* = 0.027; HR vs C, *p* < 0.001; HR vs R, *p* = 0.047), but there was no significant difference between group R and group C. Among the three groups of LR, MR and HR, the MR group had the highest mean value, and the order of magnitude was MR > LR > HR, but there was no statistically significant difference among the three groups. (**Figure 2D**)

### 3.3 Skeletal microstructure of rats in each group

**Figure 3:**
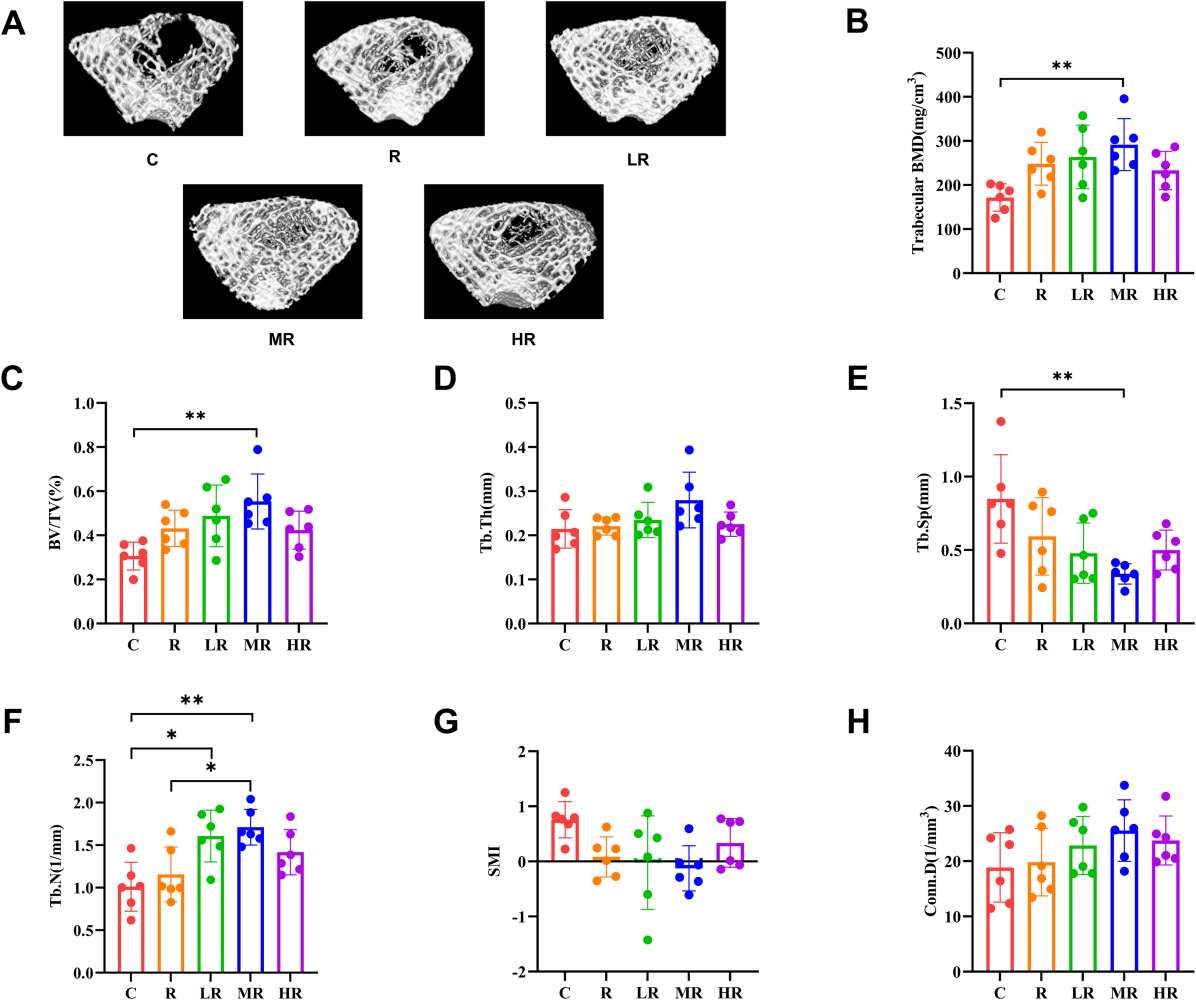
**(A)** Micro-CT three-dimensional reconstruction of bone trabeculae in each group of rats. **(B)** The effect of 8-week exercise intervention on the bone trabecular density of rats. **(C)** The effect of 8-week exercise intervention on bone volume fraction in rats. **(D)** The effect of 8-week exercise intervention on the thickness of bone trabeculae in rats **(E)** The effect of 8-week exercise intervention on the separation degree of bone trabeculae in rats. **(F)** The effect of 8-week exercise intervention on the number of bone trabeculae in rats. **(G)** The effect of 8-week exercise intervention on the trabecular bone structure mode index of rats. **(H)** The effect of 8-week exercise intervention on the density of bone trabecular connections in rats. (*indicates *p*<0.05; **indicates *p*<0.01).

**Figure 3A** shows representative three-dimensional micro-CT reconstruction images of trabecular bone from rats in each group. The results of Micro-CT test showed that the trabecular bone mineral density of the MR group was significantly different from that of the C group (η^2^ = 0.410, *p* = 0.008, F = 4.340; MR vs C, *p* = 0.006) after 8 weeks of training, and the other groups were also different from the C group, but the difference was not significant (**Figure 3B**). According to the **Figure 3C**, it can be observed that the bone volume fraction of the MR group was significantly higher than that of the C group (η^2^ = 0.431, *p* = 0.006, F = 4.729; MR vs C, *p* = 0.003), which was statistically different. Compared with group C, the bone volume fraction of other groups also increased, but the difference was not statistically significant. The results of trabecular bone separation showed that all exercise groups had changes compared with group C, but only the MR group had statistical difference (η^2^ = 0.432, *p* = 0.005, F = 4.759; MR vs C, *p* = 0.003) (**Figure 3E**). The number of trabeculae in the LR group was significantly different from that in the C group and the number of trabeculae in the MR group was significantly different from that in the C and R groups (η^2^ = 0.517, *p* = 0.001, F = 6.680; LR vs C, *p* = 0.011; MR vs C, *p* = 0.002; MR vs R, *p* = 0.021) (**Figure 3F**). However, the results of the three indicators, trabecular thickness (**Figure 3D**), differences in trabecular structural mode index (**Figure 3G**), and trabecular connection density (**Figure 3H**), showed no statistically significant differences in the exercise group despite their advantages.

### 3.4 Biomechanical properties of rats in each group

**Figure 4:**
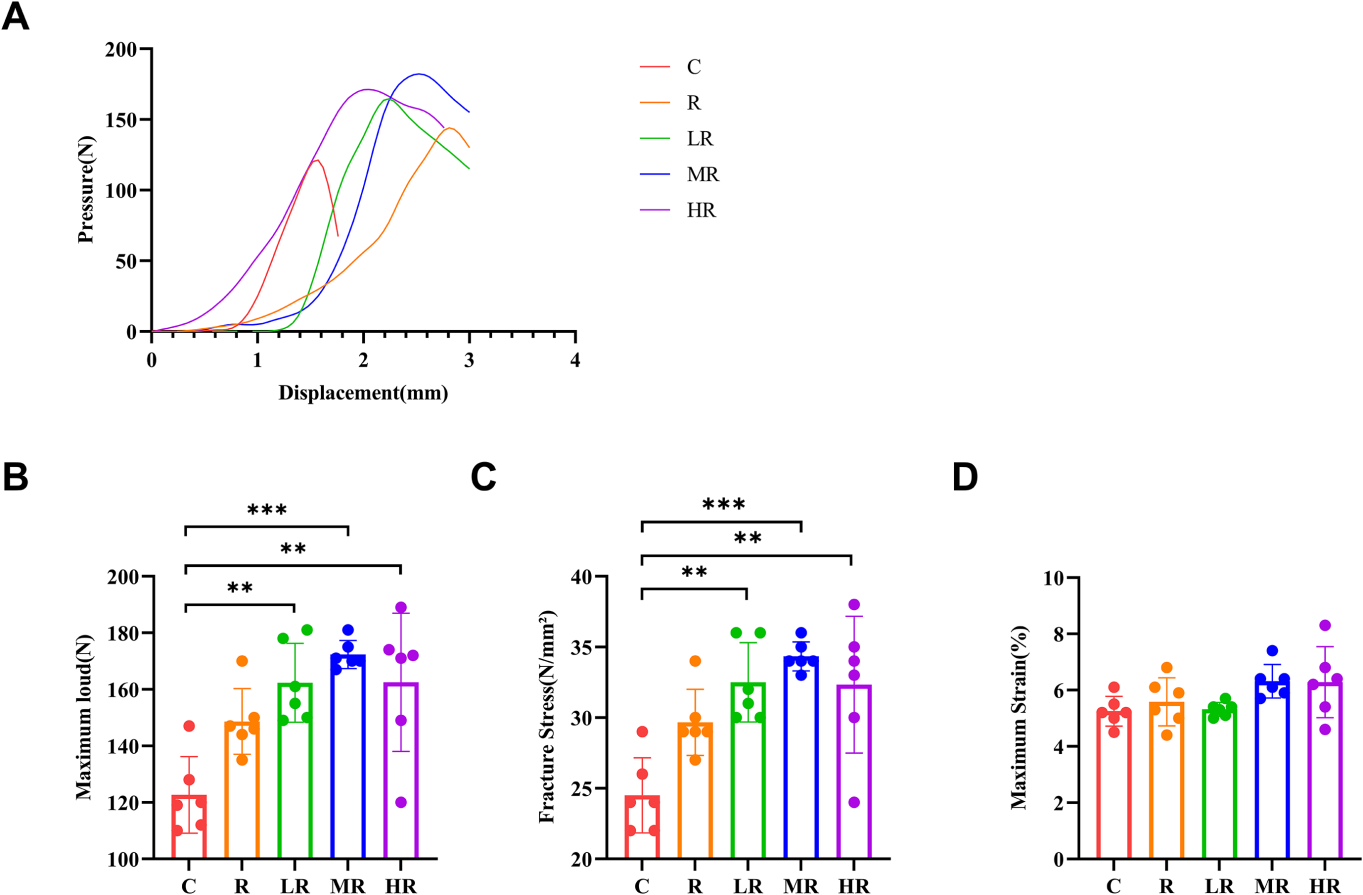
**(A)** Representative graphs of the three-point bending test of the femur in different groups. **(B)** The effect of 8-week exercise intervention on the maximum load of the femur in rats. **(C)** The effect of 8-week exercise intervention on the stress of femoral fractures in rats. **(D)** The effect of 8-week exercise intervention on the maximum strain of the femur in rats. (**indicates *p*<0.01; ***indicates *p*<0.001).

The results of biomechanical indexes of bone in each group are shown in **Figure 4**. Maximum load (η^2^ = 0.611, *p*<0.001, F = 9.823; LR vs C, *p* = 0.001; MR vs C, *p* < 0.001; HR vs C, *p* = 0.001), fracture stress (η^2^ = 0.610, *p*<0.001, F = 9.769; LR vs C, *p* = 0.001; MR vs C, *p* < 0.001; HR vs C, *p* = 0.001) were significantly higher in the LR, MR and HR groups than in the C group. However, there was no significant difference in the maximum strain index among the five groups.

### 3.5 Bone turnover markers of rats in each group

**Figure 5:**
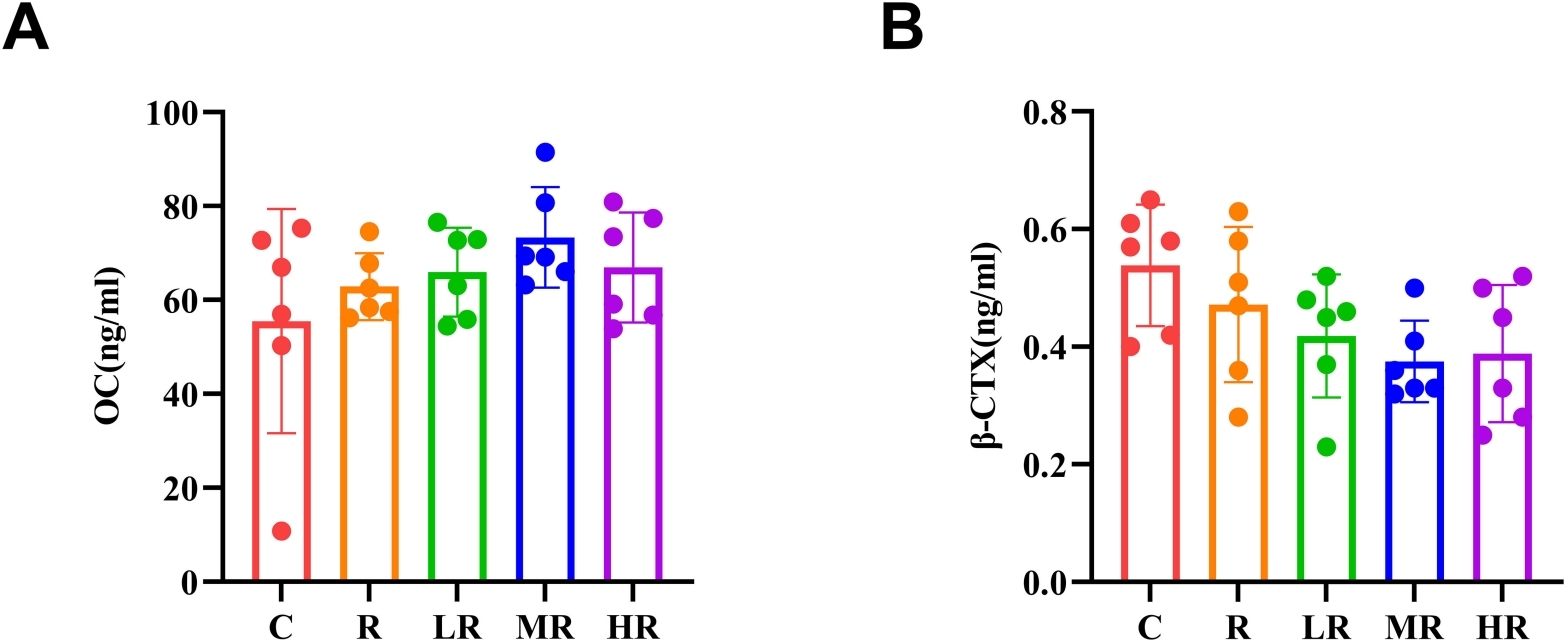
**(A)** The effect of 8-week exercise intervention on osteocalcin concentration in rats. **(B)** The effect of 8-week exercise intervention on β-CTX concentration in rats.

After eight weeks of training, the bone turnover markers of rats in each group were shown in **Figure 5**. Our results showed that the concentration of OC in the MR Group was higher than that in the other groups, but the difference was not statistically significant (*p* > 0.05). The concentration order was MR > HR > LR. As for the markers of bone resorption, the concentration of β-CTX in MR group was lower than that in other groups, but there was no statistical significance (*p*> 0.05). The concentration order was MR < HR < LR.

## 4 Discussion

Previous research suggests that exercise type and intensity exert independent and additive effects on bone mineral density. Both endurance training and resistance training have been shown to increase bone mineral density. And previous studies have shown that the combination of endurance training and resistance training has a significant effect on bone mineral density compared with endurance training or resistance training alone. However, there is controversy regarding the choice of endurance intensity. On the one hand, some studies have shown that moderate intensity endurance training is superior to high intensity and low intensity endurance training in increasing bone mass (32). Other studies have suggested that high-intensity interval training has more advantages in increasing bone mass (33).

However, comparative studies examining the effects of combining resistance training with endurance training at different intensities are lacking. Therefore, the present study investigated the effects of different intensity endurance combined with resistance training on bone mineral density, bone microstructure, bone mechanical stress, and bone-related serum indexes in adolescent male rats. Our results suggested that moderate-intensity endurance combined with resistance exercise was most effective in increasing bone mineral density, bone microarchitecture such as trabecular number, and bone peak load in rats.

### 4.1 Effects of different intensity endurance combined with resistance training on body weight and body fat percentage in rats

Body weight can reflect the growth and health status of the rat, and regular measurement of the rat’s body weight can help us assess whether the rat is adapted to the training protocols. Body fat percentage can reflect the degree of obesity, health status and metabolic risk. From our results, the body weight of the rats in each group showed a steady increasing trend over time, and from the second week, the body weight of the rats in group C was higher than that in the exercise group. At the end of the exercise intervention, rats in group C had the highest mean body weight. In our study, although the weight of the rats in the exercise intervention group was lower than that in the C group, there was no significant difference between the different intensity endurance combined with resistance exercise groups. However, in the result of body fat percentage, there were significant differences between the exercise group and the C group.

In a human study by Campos (22), it was found that endurance combined with resistance training resulted in significant reductions in body weight (kg), BMI, total fat (kg and %), and central, visceral, and subcutaneous fat (cm) in obese adolescents. The results of Nayoung (34) proved that endurance combined with resistance training can reduce the body weight of normal middle-aged rats and rats guided by high-fat diet. Our results showed that combined endurance and resistance training could reduce body weight and prevent obesity in growing rats.

### 4.2 Effects of different intensity endurance combined with resistance training on bone mineral density in rats

As a key indicator of bone mineral content per unit volume, bone mineral density can accurately and sensitively reflect the bone health status of individuals, and is an indispensable ruler for evaluating bone growth and development (35). From our results, all three combined training groups (LR, MR, and HR) showed statistically significant improvements compared to control (C) and resistance-only (R) groups. Although MR showed a slightly more favorable trend in bone mineral density, no statistically significant differences were observed among the different combined training intensity groups. This trend is worthy of attention, but further research is needed to confirm it. This indicates that endurance combined with resistance training has more advantages in increasing bone mineral density than resistance training alone and no exercise group, which is consistent with the study of Wang and Campos (22) et al.

### 4.3 Effects of different intensity endurance combined with resistance training on bone microstructure in rats

Nilsson’s (36) research suggests that the benefits of exercise for bone growth may result from changes in bone geometry and spatial structure. The mechanism of exercise on bone microstructure may be that mechanical stimulation triggers adaptive remodeling of bone, which optimizes micro-parameters such as trabecular bone arrangement, cortical bone thickness and porosity, thereby improving bone mechanical properties and damage resistance.

Our results showed that the bone volume fraction was higher in all exercise groups than in the C group, but only the MR group showed a significant change. Trabecular bone separation was less in all exercise groups than in group C, but only the MR group showed a significant change. The number of bone trabecular in all exercise groups was higher than that in group C, and significant changes were found in LR and MR groups, and there were also significant differences between group MR and group R. There were changes in trabecular thickness, trabecular structural pattern, and trabecular connection density in all exercise groups compared with group C, but none were statistically significant.

Our findings are consistent with those of Bourrin’s study (37). In both our studies, we observed that although both moderate-intensity and high-intensity treadmill training led to changes in the spatial organization of trabecular bone in the epiphyseal region, the moderate-intensity group showed a greater increase in bone volume fraction compared to the high-intensity group. This may be why the moderate-intensity endurance combined with resistance training group had an advantage in increasing trabecular bone number. Although resistance exercise was not combined in these studies, we speculate that the reasons for these results are similar: overtraining is harmful to bone, and low training intensity may not be sufficient to produce functional adaptations (38). Our research indicates that in terms of optimizing bone microstructure, moderate-intensity endurance combined with resistance exercise may be a more advantageous option.

### 4.4 Effects of different intensity endurance combined with resistance training on bone mechanical stress in rats

While fracture risk correlates with decreased bone mineral density, fractures can occur in individuals with normal bone mineral density, indicating that bone strength encompasses multiple components beyond density (39). Therefore, three-point bending test was used to perform destructive mechanical testing to obtain the ultimate mechanical parameters, which could directly quantify the resistance of bone to deformation and fracture. Animal studies have shown that low to moderate intensity treadmill training can significantly increase the fracture load, thickness and elastic modulus of the cortical bone of the femur and tibia in rats (40–42). Clarisa (43) confirmed that high-intensity treadmill training improved bone strength in rats by enhancing mineralization and hardness. However, sustained high-intensity endurance training may impair peak bone mass in young beagles (44). In Emanuel’s (45) study, it was found that high intensity interval training had a negative effect on bone toughness in healthy mice.

Our results showed that peak load and fracture stress in LR, MR, and HR groups were significantly higher than controls. The mean value of the MR group was higher than that of the LR and HR groups, but the difference was not statistically significant. The maximum strain was higher in all exercise groups than in group C, but the difference was not statistically significant. Our research indicates that endurance training combined with resistance training has a significant advantage in increasing mechanical stress on bones.

### 4.5 Effects of different degrees of endurance combined with resistance training on bone serum markers in rats

Bone is a dynamic tissue that requires continuous remodeling to prevent the accumulation of bone damage, maintain its mechanical strength, and maintain calcium homeostasis (46). During bone remodeling, bone resorption by osteoclasts occurs first, followed by bone formation by osteoblasts. These processes are balanced by coupling to ensure precise formation of new bone at the site of resorption, thereby maintaining bone quality and strength throughout the lifespan (47).

Therefore, in our study, serum bone resorption marker and bone formation marker osteocalcin were measured to reveal the response of bone metabolism to different intensity endurance combined with resistance training. Our results showed that osteocalcin concentrations in serum bone formation markers were higher in all exercise groups than in group C, but no statistical difference was observed. In serum bone resorption markers, β-CTX concentrations were lower in all exercise groups than in group C, but were not statistically different from group C. Although there was no statistical difference in this measure, the exercise group all had higher osteocalcin concentrations and lower β-CTX concentrations than the C group, while the MR group had the highest osteocalcin concentrations and the lowest β-CTX concentrations. According to Hou’s (48) review, exercise intensity and duration are important factors affecting bone metabolism. Excessive intensity or prolonged exercise will be detrimental to bone metabolism, while high-intensity exercise is beneficial to bone metabolism in the short term. However, in our study, we did not observe significant differences in serum biomarkers. We speculate that the lack of significant differences could be due to the inherent biological variability of these markers and the relatively small sample size, which may have limited the detection of subtle differences. More importantly, from a biological perspective, this finding may suggest that the endurance combined with resistance exercise protocol in this study effectively promotes bone structural adaptation (as shown by the increase in bone density) without negatively impacting overall bone metabolism.

In summary, this study demonstrates that moderate endurance combined with resistance training (MR) is superior to other exercise groups in increasing bone density, optimizing bone structure, and enhancing bone strength. Bone response to mechanical loading follows an inverted U-shaped or threshold curve. Based on the magnitude of mechanical strain, bone loading can be categorized into four windows: disuse, physiological, mild overuse, and damage (49). The superior response of the MR group may result from its precise placement in the optimal “physiological window.”

Compared to the LR group, the MR group provided significantly higher mechanical loading, which surpassed the critical threshold necessary to activate bone formation. This loading more effectively stimulates bone cell signaling pathways (e.g., Wnt/β-catenin) and promotes bone matrix mineralization. In contrast, the HR group, while delivering high loading, may have induced negative physiological stress due to overtraining, such as elevated catabolic hormones (e.g., cortisol), excessive oxidative stress, or microdamage (50). These factors could counteract the anabolic effects of mechanical loading and disrupt normal bone remodeling.

The MR group, therefore, strikes an optimal balance between mechanical loading, muscle coordination, and systemic metabolic support, effectively driving adaptive bone remodeling, which results in both bone mass accumulation and microstructure optimization.

### 4.6 Limitation

Several limitations should be acknowledged in this study. First, the sample size in each group was relatively small (n = 6). Although this sample size is consistent with that commonly used in rodent exercise intervention studies and was sufficient to detect group differences in the primary outcomes, it may have limited the statistical power for variables with greater inter-individual variability. Future studies with larger sample sizes are warranted to enhance the robustness and generalizability of the findings. Second, only male rats at a single pubertal stage were included. While this approach minimized hormonal variability, it limits the extrapolation of the results to females and to other developmental stages. Future investigations incorporating both sexes and multiple age groups will be necessary to better define sex- and age-specific skeletal responses to exercise. Third, the absence of an endurance-only training group limits our ability to isolate the independent contribution of endurance exercise to the observed skeletal adaptations. Future studies including such a group are needed to further clarify the synergistic and additive effects of combined endurance and resistance training. Fourth, although serum markers of bone metabolism were assessed, dynamic measures of bone formation and resorption, as well as histomorphometric analyses, were not performed. In addition, micro-CT analyses primarily focused on trabecular bone, without detailed assessment of cortical bone microstructure, and skeletal outcomes were evaluated only in the femur. Therefore, potential site-specific responses and cortical-level adaptations could not be fully characterized. Finally, osteocyte-specific markers (e.g., sclerostin) were not measured, which limits direct molecular confirmation of osteocyte-mediated mechanotransduction. Future studies integrating cellular, molecular, and tissue-level analyses will be important to further elucidate the mechanisms underlying exercise-induced skeletal adaptation.

## 5 Conclusion

Exercise intervention during adolescence can effectively improve bone mineral density. Compared with resistance training alone, combined endurance and resistance training shows a more favorable overall trend in improving bone-related outcomes.

Among the combined training protocols, moderate-intensity endurance training combined with resistance training exhibits the most consistent pattern of improvement in bone mineral density, bone microarchitecture, and mechanical properties, suggesting that it may represent a more favorable loading intensity range for skeletal adaptation during adolescence.

## Data Availability

The original contributions presented in the study are included in the article/supplementary material. Further inquiries can be directed to the corresponding author.
